# Evaluating the Use of Serum Inflammatory Markers for Preoperative Diagnosis of Infection in Patients with Nonunions

**DOI:** 10.1155/2017/9146317

**Published:** 2017-10-10

**Authors:** Song Wang, Peng Yin, Chenliang Quan, Kamran Khan, Guoqi Wang, Lijuan Wang, Lin Cui, Licheng Zhang, Lihai Zhang, Peifu Tang

**Affiliations:** ^1^Department of Orthopaedics, Chinese PLA General Hospital, No. 28 Fuxing Road, Beijing 100853, China; ^2^Medical College, Nankai University, No. 94 Weijin Road, Tianjin 300071, China; ^3^Department of Orthopedics, Beijing Chaoyang Hospital, China Capital Medical University, Beijing 100020, China; ^4^Bioengineering Laboratory, Department of Orthopedic Surgery, Massachusetts General Hospital and Harvard Medical School, Boston, MA, USA; ^5^Department of Chinese Medicine, Chinese PLA General Hospital, Beijing 100853, China; ^6^Anesthesia Surgery Center, Chinese PLA General Hospital, No. 28 Fuxing Road, Beijing 100853, China

## Abstract

**Purpose:**

The aim of this study is to evaluate the effectiveness of laboratory serum tests in the diagnosis of infected nonunion.

**Methods:**

Forty-two patients suspected of having infected nonunion were investigated in the study. The serum levels of white blood-cell count (WBC), C-reactive protein (CRP), erythrocyte sedimentation rate (ESR), and interleukin-6 (IL-6) were measured. A positive diagnosis of infection was made on the basis of the positive culture results. The sensitivity, specificity, positive predictive value (PPV), and negative predictive value (NPV) of each test were calculated.

**Results:**

The sensitivity and specificity of CRP both were higher than IL-6: 60.0% versus 57.1% and 85.7% versus 57.1%, respectively. With one, two, three, and four positive tests, the predicted probabilities of infection were 66.7%, 90.9%, 100%, and 100%, respectively, but the number of patients who had three or four positive tests was small.

**Conclusions:**

The diagnostic utility of IL-6 is inferior to CRP and the finding conflicts with previous conclusions drawn from periprosthetic infections. Laboratory analysis of serum inflammatory markers alone is not an effective screening tool for patients suspected of having an infected nonunion.

## 1. Introduction

Infected nonunion of fractures remains an expensive and a devastating complication that can present pain, swelling, erythema, and a draining sinus. Evaluating these clinical parameters is the first and foremost step in the diagnostic procedure for a nonunion patient with suspected infection. However, patients with typical clinical manifestations of infection are decreasing, while quiescent infections are rising in recent years [1]. Low virulence bacteria and “biofilm pathogens” surrounding the implant have been reported as the primary causes that induce indolent infection [2]. Use of antibiotics is a standard protocol for most fracture treatments but infection is still highly prevalent. There is no standardized method for diagnosing infection prior to nonunion surgery and making an accurate discrimination of infection from aseptic nonunions is difficult.

The therapeutic strategy of infected nonunion is distinctively different from aseptic nonunion. The treatment of infected nonunion often needs multiple stages, including debridement of infected tissues and application of external fixation [3]. In contrast, aseptic nonunion can be surgically treated in a single stage with bone grafting and replacement of internal fixation [4, 5]. Therefore, the surgeon would like to know whether the nonunion is infected or aseptic prior to the operation. Reliable results of examinations (including laboratory tests, imaging modalities, and intraoperative cultures) are extremely important for proper diagnosis. Among these tests, intraoperative culture is regarded as the gold standard for the diagnosis of infected nonunions. However, intraoperative culture requires long culturing time of pathogens and suffers from relatively inadequate sensitivity [6]. Laboratory tests (including white blood-cell count [WBC], C-reactive protein level [CRP], and erythrocyte sedimentation rate [ESR]) have been used to examine the risk of a positive culture.

The use of biomarkers is advantageous because they are simple to administrate and less time consuming. They have been successful in properly evaluating musculoskeletal infection [7]. Interleukin-6 (IL-6) is a newer serum inflammatory marker that has been successfully evaluated in multiple papers of periprosthetic infections. Literature has recognized the diagnostic utility of IL-6 since it is superior to the traditional serum markers [8–10]. IL-6 is secreted by various cell types, such as T cells, B cells, osteoblasts, and macrophages [11]. As a regulator of immune response and acute phase reactions, the concentration of IL-6 increases more rapidly and returns to normal more quickly than CRP and ESR [12, 13] and has been predicted to be a potential serum indictor of infected nonunion. Little is known about the diagnostic performance of serum IL-6, in septic nonunion [7]. Due to lack of sufficient evidence, the test remains outside the standard diagnostic protocol for the detection of infected nonunion.

The purpose of this study was to investigate the diagnostic value of the preoperative laboratory protocol including IL-6, WBC, CRP, and ESR in bone nonunion patients with suspected infection.

## 2. Materials and Methods

This study was approved by our institutional review board. Patients were retrospectively identified for the present study. Between January 2014 and January 2016, 123 nonunion patients were treated operatively. Overall 81 patients were excluded because of administration of antibiotics before surgery, unavailability of complete examinations, or another reason according to the criteria presented below. The average age of the 42 enrolled patients (32 males and 10 females) was 39 years (range, 18 to 68). The locations of nonunions were as follows: three humeral nonunions, thirteen femoral nonunions, twenty-one tibial nonunions, two radius nonunions, and three foot nonunions.

The definition of a long-bone nonunion was “radiographic evidence of nonprogression of healing for at least 3 months, or lack of healing by 9 months since the initial injury” [14–16]. To identify an infection, intraoperatively, multiple gross tissue specimens (at least five samples) were cultured. A positive diagnosis of infection was made if there was at least two same positive growth types on culture of the intraoperative samples. Only high-risk patients, that is, a history of open fracture or any infection signs (erythema, warmth, and draining sinus), were initially included and the crucial clinical data was carefully collected. Inclusion criteria were a diagnosis of nonunion and complete examinations including WBC, CRP, ESR, IL-6, and bacteria culture. Patients with sepsis or infections not involving the fracture site were excluded.

Antibiotic use for the participants was delayed until after intraoperative specimens were collected, unless the patient needed anti-infective therapy urgently and then these patients were excluded. Blood samples for laboratory analysis were obtained immediately after entering the hospital. Positive results of laboratory tests were defined by the reference values in our hospital laboratory: a WBC count of >10 × 10^9^/L, an ESR of >20 mm/h, a CRP of >0.8 mg/dl, and IL-6 level of >5.9 pg/ml. During surgery, surgical specimens (abnormal soft tissue, “limy membrane,” and pus liquid) were aseptically collected from the location surrounding the implants and these gross specimens (at least five samples) were immediately sent to microbiology and pathology laboratory for cultures.

## 3. Statistical Analysis

The sensitivity, specificity, positive predictive value (PPV), and negative predictive value (NPV) of each test were calculated. Sensitivity was defined as the number of true positives divided by (number of true positives + number of false negatives). Specificity was defined as the number of true negatives divided by (number of true negatives + number of false positives). Positive predictive value (PPV) was defined as Σ true positive/Σ test outcome positive and negative predictive value (NPV) was defined as Σ true negative/Σ test outcome negative. Lastly, 95% confidence intervals were calculated according to the efficient-score method [17].

## 4. Results

Overall, forty-two patients (32 males and 10 females) were reviewed in the study with an average age of 39 years (range, 18 to 68). All individuals were followed to either bone healing or infection.

Thirty-five patients were confirmed as having infected nonunions according to their positive culture results. Nineteen patients were positive for* Staphylococcus aureus*, six patients were positive for* Escherichia coli*, two patients were positive for* Staphylococcus epidermidis*, two patients were positive for* Enterobacter cloacae*, one patient was positive for* Enterococcus faecalis*, and five patients grew both* S. aureus* and* E. coli*. Among the thirty-five patients, twelve had shown signs of infection: seven patients complained of discharging pus, four patients presented with chronic sinus tract, and one patient suffered disorder of incisional wound involving recurrent swelling and erythema. Additionally, one patient had intraoperative infection evidence (pus liquid) at the nonunion site but specimen cultures were negative (case number 20, Table 1).

The sensitivity and specificity of each test are listed in Table 2. The white blood-cell count (WBC) had high specificity (85.7%, 95% CI: 42.01–99.25) but lowest sensitivity (22.9%, 95% CI: 11.04–40.55). Similarly, the ESR also had high specificity (71.4%, 95% CI: 30.26–94.89) but low sensitivity (37.1%, 95% CI: 21.99–55.05). In addition, the sensitivity and specificity of CRP were both higher than the serum IL-6: 60.0% versus 57.1% and 85.7% versus 57.1%, respectively.

The probability of a participant having infection was analyzed as a function of the number of positive serum markers (Table 3). With one, two, three, and four positive tests, the predicted probabilities of infection were 66.7%, 90.9%, 100%, and 100%, respectively. However, the number of patients who had three or four positive tests was small, 9.5% and 14.3%, respectively. Therefore, two positive serum markers among the four tests were the relatively effective point, which had the high accurate predictability of infection and relatively high rates among infected nonunion patients. When combining the positive results of any two tests, we found the probability of infection was 100% (except CRP + IL-6 and IL-6 + ESR) but with low probability of occurrence (the rate among infected nonunion patients was CRP + ESR: 25.7%; CRP + WBC: 17.1%; IL-6 + WBC: 20%; WBC + CRP: 11.4%). The probability of IL-6 + ESR was 90.9% with the occurrence rate of 28.6%. The probability of IL-6 + CRP was 93.8% with the occurrence rate of 42.9%. It should be noted that even if all laboratory tests were negative, there was still 77.8% probability of infection.

## 5. Discussion

The diagnosis of infected nonunion is a challenging, and there has not been a preoperatively estimated standard protocol. Although a variety of studies on preprosthetic infections have shown that IL-6 was a significant independent predictor of infection [8, 10], there is little knowledge about the utility of IL-6 in the diagnosis of infected nonunion. Hence, we integrated IL-6 into traditional diagnostic protocol including WBC, CRP, and ESR. Our data demonstrated that IL-6 had a poor sensitivity and specificity in detection of infected nonunions and was inferior to the traditional test of CRP, 57.1% versus 60.0% and 57.1% versus 85.7%, respectively.

IL-6 as a new serum inflammatory marker has been demonstrated to have distinct advantages for the detection of acute musculoskeletal infections [8, 9, 18]. For instance, Wirtz et al.'s research showed that IL-6 was superior to traditional serum markers in the diagnosis of hip and knee preprosthetic infections [13]. However, there was also an opposite opinion. Villacis et al. believed that IL-6 was not a superior diagnostic method for infection compared with the basic test [19]. In our study, our results suggested IL-6 was not an effective diagnostic test for infected nonunion; that is, the sensitivity and specificity of CRP which are basic tests for the investigation of septic nonunion were both higher than IL-6. We thought that the different diagnosis time points might be the main reasons for the different results. The concentration of IL-6 increases more rapidly and returns to normal more quickly than CRP and ESR [12, 13]. Nonetheless, the response of IL-6 is often dulled in chronic infections due to its rapid return to normal levels [20]. According to the definition of a nonunion mentioned above [14–16], patients with infected nonunions usually suffered from long-term infection. The average time of a patient from initial injury to investigation in the study was 28 months and we think this explains why IL-6 has a limited utility for detection of infected nonunion in our study.

Stucken et al. [7] have reported that the cumulative use of biomarkers had a reliable predicted probability of infection among nonunion patients. In this paper, we first integrated IL-6 into our protocol to evaluate its diagnostic value. In this study, if a patient had three or four positive laboratory tests, the predicted probability of infection was 100%. However, the number of patients who had three or four positive tests was rarely found in clinical practice, 9.5% and 14.3% respectively. The combination value of any two biomarkers was further explored and we found that the predicted probabilities of the protocol showed high detection accuracy, but the number of patients who had two positive tests was relatively small. Additionally, even when all the tests were negative, 77.8% of patients were finally diagnosed as having septic nonunion. This shows positive laboratory tests are useful for ruling in but not ruling out infections.

This present study has several limitations. First, the number of patients examined in our study was relatively small and there were very few individuals in the negative culture group. In our hospital, only high-risk patients were determined by bacteria culture so that is one of the reasons why more patients with positive culture are observed in the study. Second, the pathology results of involved patients were not available. We cannot get more information about the diagnostic value of pathology results which is an important tool for diagnosis of infection [21, 22]. Another limitation is that some test data of CRP and IL-6 provided by our hospital laboratory was in interval value form (e.g., the data of IL-6 of a patient was described as “IL-6 level < 2.00 pg/ml”) and the distributions of variables were not acquired. Therefore, we cannot add receiver operating characteristic (ROC) analysis in the study.

Our data indicates that the diagnostic accuracy of IL-6 is not superior to CRP and laboratory analysis of serum inflammatory markers alone is not an effective screening tool for patients suspected with infected nonunion. Inflammatory markers including IL-6 do not allow reliably diagnosing implant-associated osteomyelitis in patients with nonunion. The results from the present study can help surgeons in managing risk and making more informed decisions about patients' healthcare.

## Figures and Tables

**Table 1:** Summary of patient characteristics.

Case number	Age	Sex	Fracture site	Previous treatment	Culture
1	45	Male	Humerus	Plate, screws	*S. aureus*
2	21	Male	Femur (open)	Plate, screws	*S. aureus*
3	53	Female	Tibia	Plate, screws	Neg
4	32	Male	Femur	Plate, screws	*E. coli*
5	48	Female	Foot	Screws, external fixator	*S. aureus*
6	48	Male	Femur (open)	Plate, screws	*S. aureus*, *E. coli*
7	54	Female	Tibia	Plate, screws	*Enterococcus faecalis*
8	24	Male	Tibia	Plate, screws	Neg
9	26	Male	Tibia	Nail	*S. aureus*
10	58	Male	Tibia	External fixator	*E. coli*
11	50	Female	Tibia	Plate, screws	*S. aureus*
12	37	Male	Tibia	External fixator	*S. aureus*
13	44	Male	Femur	Plate, screws	*S. epidermidis*
14	41	Female	Tibia	Plate, screws	*S. aureus*
15	38	Male	Tibia	Plate, screws	*S. aureus*
16	29	Male	Femur	Nail	Neg
17	38	Male	Femur	Nail	S. aureus
18	44	Female	Tibia	Plate, screws	*S. aureus*, *E. coli*
19	27	Male	Tibia (open)	Plate, screws	*S. aureus*, *E. coli*
20	32	Male	Femur	Plate, screws	Neg
21	23	Female	Femur	External fixator	*E. coli*
22	18	Male	Humerus	Plate, screws	*S. aureus*
23	41	Male	Femur	Nail	*S. aureus*
24	44	Male	Humerus	Plate, screws	Neg
25	68	Male	Femur	Plate, screws	*S. aureus*
26	32	Male	Tibia	Plate, screws	Neg
27	45	Male	Tibia (open)	External fixator	*S. aureus*
28	42	Male	Tibia (open)	Plate, screws	*E. cloacae*
29	48	Male	Femur	External fixator	*S. aureus*, *E. coli*
30	24	Female	Tibia (open)	Plate, screws	*S. aureus*
31	36	Female	Foot	Screws, external fixator	*E. coli*
32	38	Male	Tibia	Plate, screws	*S. aureus*
33	58	Male	Tibia (open)	Plate, screws	*S. aureus*, *E. coli*
34	47	Male	Tibia	Plate, screws	*S. aureus*
35	19	Female	Radius	Plate, screws	*S. aureus*
36	53	Male	Tibia (open)	External fixator	*E. coli*
37	32	Male	Tibia (open)	Plate, screws	*S. aureus*
38	30	Male	Foot	Plate, screws	*S. epidermidis*
39	50	Male	Tibia	Plate, screws	*S. aureus*
40	36	Male	Radius (open)	Plate, screws	Neg
41	41	Male	Femur (open)	External fixator	*E. coli*
42	43	Male	Femur	Plate, screws	*E. cloacae*

**Table 2:** Sensitivity, specificity, and positive and negative predictive values of each test.

Test	Sensitivity (95% CI)	Specificity (95% CI)	Positive predictive value (PPV) (95% CI)	Negative predictive value (NPV) (95% CI)
WBC	22.9% (11.04–40.55)	85.7% (42.01–99.25)	88.9% (50.67–99.42)	18.2% (7.62–36.08)
CRP	60.0% (42.21–75.65)	85.7% (42.01–99.25)	95.5% (75.12–99.76)	30.0% (12.84–54.33)
IL-6	57.1% (39.52–73.24)	57.1% (20.24–88.19)	87.0% (65.33–96.57)	21.1% (6.97–46.10)
ESR	37.1% (21.99–55.05)	71.4% (30.26–94.89)	86.7% (58.39–97.66)	18.5% (7.03–38.75)

95% CI: 95% confidence interval.

**Table 3:** Predicted probability of cumulative use of all tests.

Number of positive tests under consideration	Number of patients	Predicted probability of infection (%)
4	4 (9.5%)	100%
3	6 (14.3%)	100%
2	11 (26.2%)	90.9%
1	12 (28.6%)	66.7%
0	9 (21.4%)	77.8%

The predictors include WBC, IL-6, CRP, and ESR level.
